# Regional Transmission of *Salmonella* Paratyphi A, China, 1998–2012

**DOI:** 10.3201/eid2305.151539

**Published:** 2017-05

**Authors:** Xin Lu, Zhenpeng Li, Meiying Yan, Bo Pang, Jialiang Xu, Biao Kan

**Affiliations:** State Key Laboratory of Infectious Disease Prevention and Control, Chinese Center for Disease Control and Prevention, Beijing, People’s Republic of China (X. Lu, Z. Li, M. Yan, B. Pang, J. Xu, B. Kan);; Collaborative Innovation Center for Diagnosis and Treatment of Infectious Diseases, Hangzhou, People’s Republic of China (X. Lu, Z. Li, M. Yan, Pang, B. Kan);; Beijing Technology and Business University, Beijing (J. Xu)

**Keywords:** *Salmonella enterica* serovar Paratyphi A, SNP, transmission mode, population structure, bacteria, China, single-nucleotide polymorphism

## Abstract

To explore transmission patterns and genetic relationships of *Salmonella enterica* serovar Paratyphi A in China, we conducted a genome-wide single-nucleotide polymorphism analysis on the strains in the 4 provinces in which incidence was highest during 1998–2012. Markedly phylogeographic clustering suggested regional virus circulation after introduction from areas in southeastern China.

In Asia, incidence of paratyphoid fever remains high (*1*). In the mid-1990s, the number of paratyphoid fever cases in Asia caused by *Salmonella enterica* serovar Paratyphi A started to increase (*2*–*4*). In 2000, an estimated 5.41 million cases occurred; areas where incidence was highest (i.e., >100 cases/100,000 population per year) included south-central and Southeast Asia (*5*). Since 1998, the incidence of paratyphoid fever in Asia and the world has been highest in China, ranging from 0.08 to 192.5 cases/100,000 population annually (*6*); the provinces in which incidence is highest are Guangxi, Guizhou, Yunnan, and Zhejiang (*7*).

Information about the transmission routes and risk factors for infection could be used to improve the control strategies and measures for paratyphoid fever. Laboratory-based pathogen molecular subtyping, particularly genome-wide single-nucleotide polymorphism (SNP) analysis, can markedly improve outbreak detection, source tracing, and understanding of the epidemic modes. In this study, we analyzed genome-wide SNP and epidemiologic data from *Salmonella* Paratyphi A strains isolated from the China provinces where incidence was highest over a long period (1998–2012) and detected region-limited clone expansion in the epidemic provinces.

## The Study

In 1998, the incidence of typhoid/paratyphoid fever in China was 4.82 cases/100,000 population (60,146 cases reported); this measure has since decreased annually to 0.88/100,000 (11,890 cases) in 2012 (China Information System for Disease Control and Prevention, unpub. data). Typhoid/paratyphoid fever cases in Guizhou, Yunnan, Zhejiang, and Guangxi Provinces accounted for 45.8% (in 1998) to 76.5% (in 2001) of all cases in China (Technical Appendix Figure). 

**Figure 1:**
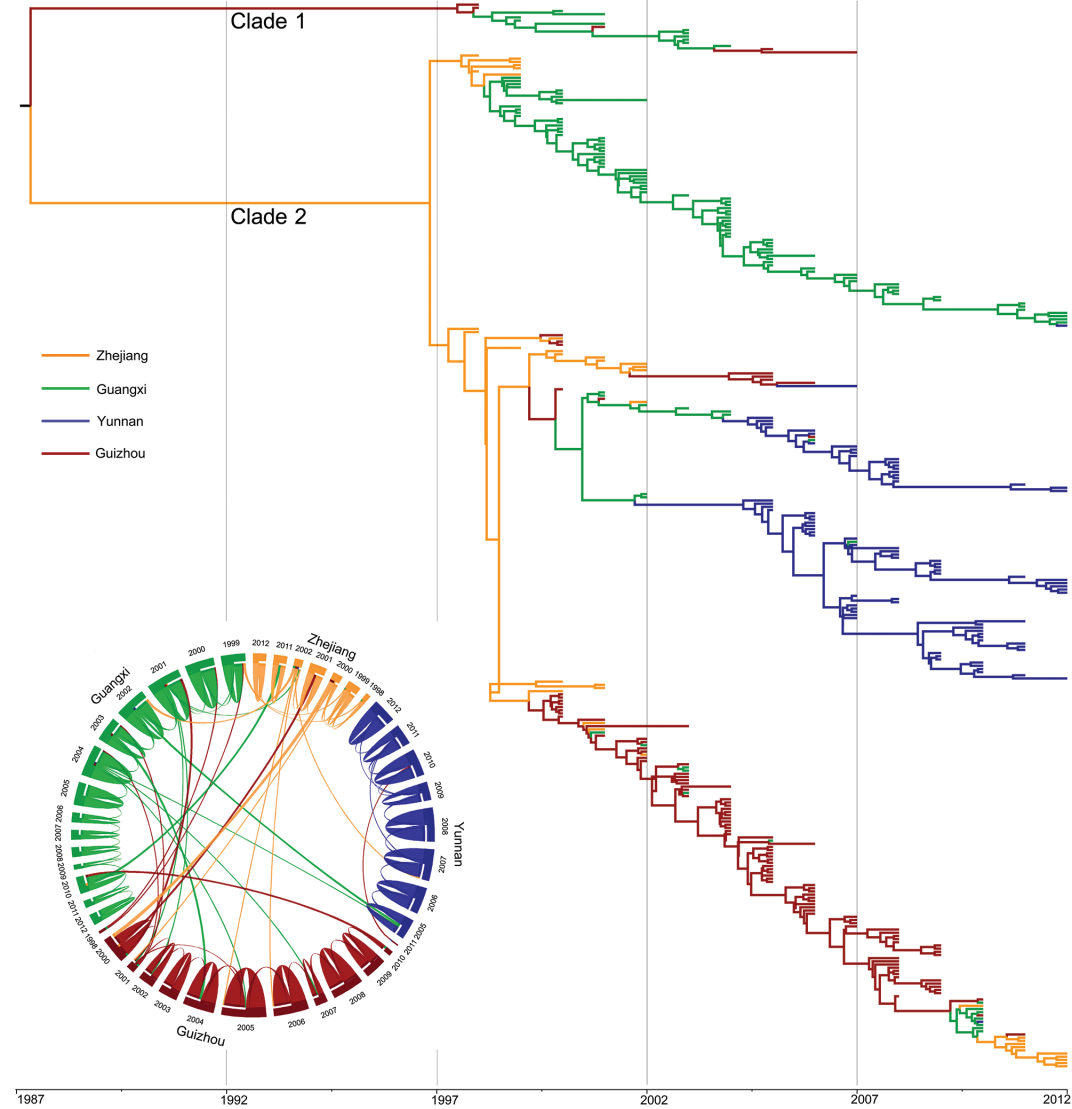
Phylogenetic tree of *Salmonella enterica* serovar Paratyphi A strains isolated from China, 1998–2012. The branches are colored according to inferred location. Inset: potential transmission of *Salmonella* Paratyphi A strains isolated from 4 provinces (Zhejiang Guangxi, Guizhou, and Yunnan). The flow bars indicate the source of transmission; 1 end of the bar directly touches the province of origin, and the other end of the bar exhibits a small gap before the province of destination.

To analyze the genomic epidemiology of paratyphoid fever in these provinces, we first selected 96 *Salmonella* Paratyphi A strains circulating in 15 provinces in China during 1998–2011 (Technical Appendix Table 1). Strains were isolated from hospitalized patients suspected of having typhoid/paratyphoid fever and were maintained in the strain bank of the Chinese Center for Disease Control and Prevention. We then conducted genome-wide SNP genotyping by using the iPLEX Gold assay (Sequenom Inc., San Diego, CA, USA) with 2,343 SNPs obtained from 7 genomes sequenced in a previous study (*8*) and 17 genomes of *Salmonella* Paratyphi A strains sequenced in this study. We obtained 112 phylogenetically informative SNPs (including 57 nonsynonymous SNPs) (Technical Appendix Table 2), which were further analyzed in 335 *Salmonella* Paratyphi A strains (Technical Appendix Table 1) isolated from the provinces where incidence was highest (i.e., Guangxi, Guizhou, Yunnan, and Zhejiang) during 1998–2012 by using the iPLEX Gold assay. The population history of *Salmonella* Paratyphi A was estimated by using BEAST version 2.1.3 (http://beast.bio.ed.ac.uk/), and the maximum clade credibility tree was summarized by using TreeAnnotator and visualized by using FigTree version 1.4.2 (both within BEAST). The consensus tree (Figure 1) showed that all strains fell into 2 main clades: clade 1 consisted of 16 strains isolated from Yunnan, Guizhou, and Guangxi Provinces during 1998–2007; clade 2 consisted of the strains that were most common and widespread in these 4 provinces during 1998–2012. In clade 2, at least 3 subclades were formed, which were markedly characterized by geographic clustering according to province (Figure 1), suggesting intraprovince transmission of the different clones. In addition, the earlier strains in the root of each major subbranch were isolated mainly from Zhejiang, and in the years before 2005, some strains from Guangxi were also mixed in the Guizhou branch.

On the basis of the trees, we further determined from/to transmission of *Salmonella* Paratyphi A by using Circos (*9*) (Figure 1). The same SNP genotypes of *Salmonella* Paratyphi A strains were preferentially transmitted within a single province from year to year, whereas the strains from Zhejiang were frequently transmitted to Guizhou and Guangxi, particularly during 1998–2002. The transmission between Guangxi and Guizhou was markedly more frequent before 2004 and decreased after 2005. After 2005, we found no transmission from Yunnan to other provinces. 

**Figure 2:**
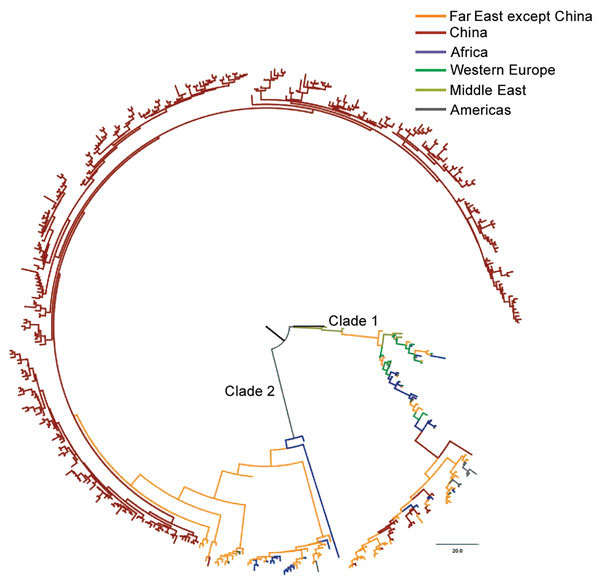
Phylogenetic tree of *Salmonella enterica* serovar Paratyphi A strains in China and worldwide. The branches are colored according to the inferred location. Scale bar indicates number of years.

We also extracted information for 112 SNPs from 127 genomes of the worldwide *Salmonella* Paratyphi A isolates in GenBank (*10*) and constructed a phylogenetic tree by combining these data with data from the 335 strains from China obtained in this study (Figure 2). The international strains fell into 2 clades, and the strains from Southeast and southern Asia were positioned much closer to the root of the strains from China, suggesting that the potential source of *Salmonella* Paratyphi A in China might be India or Indonesia.

## Conclusions

The genome-wide SNP phylogeny provided more accurate insights into the variation of *Salmonella* Paratyphi A strains in China. In Guizhou, Guangxi, and Yunnan Provinces, which are geographically adjacent, *Salmonella* Paratyphi A has existed for many years. Although we had speculated that the organism might show a mixture of genetic patterns, the phylogenetic tree showed that epidemic strains from different provinces gradually accumulated their own mutations to evolve and form obvious geographic branches. In earlier years of the study period (1998–2002), the epidemic strains from Guangxi and Guizhou Provinces might have originated from early epidemic strains from Zhejiang Province. The level of economic development in Zhejiang Province is high, whereas in Guangxi, Guizhou, and Yunnan Provinces it is lower; the rural population from these 3 provinces migrates frequently to work in the economically developed southeastern coastal areas in China, including Zhejiang (*11*), Jiangsu, and Guangdong Provinces. According to the fifth national census conducted in 2000 (http://www.stats.gov.cn/tjsj/pcsj/rkpc/5rp/index.htm) and the sixth conducted in 2010 (http://www.stats.gov.cn/tjsj/pcsj/rkpc/6rp/indexch.htm), the migration data within the 4 provinces showed this population movement trend (Technical Appendix Table 3). At irregular intervals, migrant workers, mainly those who are young and middle-aged, return to their hometown for family reunions. 

In the mid-1990s, paratyphoid fever became an emerging problem in Zhejiang Province; during 1997–2005, incidence was 8.61 cases/100,000 population (*12*). In those years, managing ex situ healthcare and medical treatments in China was problematic. When migrant workers got ill, they seldom sought medical treatment at the hospital in the city in which they worked; rather, they bought medicine at a chemist’s shop or returned to their hometown for treatment (Zhang Q. The study on the health seeking behavior of migrant workers [master’s thesis]. China: Shaanxi Normal University; 2012).

Because of lack of medical treatment in hospitals, migrant workers who become infected with *Salmonella* Paratyphi A easily become chronic carriers. Therefore, *Salmonella* Paratyphi A might be transmitted to Guangxi, Guizhou, and Yunnan Provinces via a migrating infected population, including patients and carriers. In addition, these 3 provinces are mainly mountainous, and the population flow among these provinces is limited by their lower economic development and inaccessibility. Therefore, the transmission pattern in these regions could be closely associated with the southeastern coastal areas, where the level of economic development is higher, and transmission among these 3 provinces could be absent. Moreover, in these paratyphoid-epidemic provinces, most of the overall population lives in rural agricultural areas. Given the combination of poor water and food hygiene with a hot and humid climate, the epidemic clones of *Salmonella* Paratyphi A could persist for a long time after being introduced into these areas.

In summary, we identified the evolution and transmission mode of paratyphoid fever in the China provinces where incidence is highest. Populations migrating to southeastern China probably mediated the transmission of *Salmonella* Paratyphi A. Considering the obvious regional clone expansion in these provinces, the local natural, social, and economic conditions need to be investigated for their potential roles in the spread of paratyphoid fever and for the development of intervention strategies.

Technical Appendix *Salmonella enterica* serovar Paratyphi A strains and single-nucleotide polymorphisms used in this study and incidence of typhoid/paratyphoid fever in China, 1998–2012.
