# Supplementary material for: Regional Transmission of Salmonella Paratyphi A, China, 1998–2012
Source: Emerg Infect Dis. 2017 May;23(5):833–6. doi: 10.3201/eid2305.151539 (PMC5403048; doi:10.3201/eid2305.151539)
Supplement: Technical Appendix — Salmonella enterica serovar Paratyphi A strains and single-nucleotide polymorphisms used in this study and incidence of typhoid/paratyphoid fever in China, 1998–2012. [file 15-1539-Techapp-s1.pdf]

Article DOI: http://dx.doi.org/10.3201/eid2305.151539

Regional Transmission of *Salmonella* Paratyphi A, China, 1998–2012

Technical Appendix

Technical Appendix Table 1. *Salmonella enterica* serovar Paratyphi A strains used in this study

Validation group:

Location	1998	1999	2000	2001	2002	2003	2004	2005	2006	2007	2008	2009	2010	2011	Total
Beijing	-	-	-	-	-	-	1	-	-	-	-	-	-	-	1
Fujian	-	-	-	-	-	-	-	-	2	-	-	-	-	-	2
Guangdong	-	-	2	1	1	-	-	-	-	-	-	-	-	-	4
Guangxi	-	2	1	1	1	1	2	1	1	2	-	-	1	-	13
Guizhou	1	-	3	2	3	3	3	3	3	1	2	-	-	-	24
Henan	-	-	-	-	-	-	-	-	-	2	-	-	-	-	2
Jiangsu	-	-	2	-	-	1	3	3	-	-	-	-	-	-	9
Shandong	-	-	-	-	-	-	-	2	2	-	-	-	-	-	4
Shanghai	-	-	-	-	-	-	-	-	-	-	-	-	1	1	2
Sichuan	-	-	-	-	-	-	-	-	-	-	-	1	-	-	1
Tianjin	-	-	-	-	-	-	-	-	1	-	-	-	-	-	1
Xinjiang	-	-	-	-	-	-	-	-	1	-	-	-	-	-	1
Yunnan	-	-	-	-	-	-	-	2	2	3	3	3	2	3	18
Zhejiang	3	2	3	3	3	-	-	-	-	-	-	-	-	-	14
Total	4	4	11	7	8	5	9	11	12	8	5	4	4	4	96

Test group:

Year	Guangxi (GX)	Guizhou (GZ)	Yunnan (YN)	Zhejiang (ZJ)	Total
1998	-	2	-	4	6
1999	10	-	-	6	16
2000	11	12	-	5	28
2001	15	4	-	6	25
2002	13	6	-	5	24
2003	10	8	-	-	18
2004	16	13	-	-	29
2005	9	17	8	-	34
2006	4	13	11	-	28
2007	4	5	13	-	22
2008	4	13	14	-	31
2009	2	9	8	-	19
2010	7	2	11	1	21
2011	3	1	7	5	16
2012	4	-	9	5	18
Total	112	105	81	37	335

Technical Appendix Table 2. Information on the SNPs used in the phylogenomic analysis

SNP ID	Ref position	Gene	SNP	Pos in CDS	Pos in code	REF_AA/ Query_AA	SNP type
snp_20186	5057	intergenic	A/G	NA	NA	NA	NA
snp_11428	25206	SPA0022	T/C	102	3	T/T	S
snp_11443	62331	SPA0054	T/C	1043	2	R/H	N
snp_10005	73338	SPA0064	A/G	478	1	A/T	N
snp_10009	79240	SPA0068	A/C	2443	1	K/Q	N
snp_10049	192044	SPA0163	T/C	165	3	S/S	S
snp_10096	314728	SPA0277	T/C	3	3	M/I	N
snp_20014	338428	intergenic	A/T	NA	NA	NA	NA
snp_10107	376983	SPA0335	T/C	3191	2	P/L	N
snp_10133	449853	SPA0384	A/G	183	3	T/T	S
snp_10141	471351	SPA0398	T/G	147	3	G/G	S
snp_10152	502046	SPA0433	T/G	541	1	T/P	N
snp_20025	531247	intergenic	A/G	NA	NA	NA	NA
snp_10172	549292	SPA0476	T/C	1632	3	G/G	S
snp_10174	552382	SPA0480	C/G	96	3	P/P	S
snp_10196	605878	SPA0533	A/G	50	2	I/T	N
snp_10199	611838	SPA0539	A/G	405	3	G/G	S
snp_10222	669168	SPA0592	T/C	248	2	S/F	N
snp_10245	739407	SPA0657	T/C	413	2	R/H	N
snp_10253	749350	SPA0665	T/C	519	3	P/P	S
snp_10321	921694	SPA0824	T/C	131	2	G/D	N
snp_10325	931238	SPA0835	A/G	759	3	V/V	S
snp_20045	960188	intergenic	T/C	NA	NA	NA	NA
snp_20052	1063040	intergenic	T/C	NA	NA	NA	NA
snp_1091182	1091182	SPA1020	A/G	379	1	I/V	N
snp_10432	1205933	SPA1135	T/G	218	2	L/R	N
snp_1216643	1216643	SPA1149	C/T	142	1	G/S	N
snp_30038	1259693	intergenic	A/T	NA	NA	NA	NA
snp_10481	1339722	SPA1269	A/C	216	3	R/R	S
snp_10500	1391688	SPA1310	T/C	1741	1	H/Y	N
snp_1449976	1449976	SPA1369	A/G	43	1	K/E	N
snp_10526	1457942	SPA1377	T/C	835	1	Y/H	N
snp_10541	1492951	SPA1411	A/G	137	2	W/*	N
snp_10565	1536934	SPA1463	A/G	626	2	R/K	N
snp_10569	1553735	SPA1479	C/G	888	3	A/A	S

SNP ID	Ref position	Gene	SNP	Pos in CDS	Pos in code	REF_AA/ Query_AA	SNP type
snp_10582	1588672	SPA1512	A/G	1143	3	M/I	N
snp_10590	1603642	SPA1528	A/T	256	1	T/S	N
snp_10592	1604267	SPA1529	T/C	595	1	A/T	N
snp_10594	1611049	SPA1537	T/C	539	2	G/E	N
snp_10598	1618194	SPA1543	T/C	224	2	V/A	N
snp_1629910	1629910	SPA1554	G/A	760	1	E/K	N
snp_10604	1640768	SPA1562	A/G	152	2	G/D	N
snp_10617	1678441	SPA1617	A/G	451	1	V/M	N
snp_10622	1683532	SPA1622	C/G	1312	1	P/A	N
snp_10632	1714301	SPA1649	T/G	614	2	D/A	N
snp_10637	1722697	SPA1658	A/G	183	3	R/R	S
snp_10638	1730215	SPA1666	T/C	2900	2	V/A	N
snp_10658	1787157	SPA1727	A/G	393	3	L/L	S
snp_20082	1861324	intergenic	T/G	NA	NA	NA	NA
snp_10696	1924452	SPA1848	A/G	274	1	G/S	N
snp_1937988	1937988	intergenic	C/T	NA	NA	NA	NA
snp_20089	2075885	intergenic	T/C	NA	NA	NA	NA
snp_10770	2168007	SPA2083	A/G	934	1	S/P	N
snp_10787	2211029	SPA2125	T/C	210	3	P/P	S
snp_2258461	2258461	intergenic	A/G	NA	NA	NA	NA
snp_30062	2258462	intergenic	C/G	NA	NA	NA	NA
snp_10845	2357099	SPA2264	C/G	147	3	T/T	S
snp_20107	2381065	intergenic	T/C	NA	NA	NA	NA
snp_10858	2385763	SPA2289	T/C	405	3	G/G	S
snp_10876	2424067	SPA2328	A/G	403	1	M/V	N
snp_10878	2432112	SPA2335	T/C	93	3	A/A	S
snp_10879	2433680	SPA2337	A/G	697	1	G/S	N
snp_10892	2467778	SPA2366	T/C	1944	3	L/L	S
snp_20116	2574236	intergenic	A/G	NA	NA	NA	NA
snp_2764376	2764376	SPA2658	G/A	226	1	A/T	N
snp_20128	2833595	intergenic	T/C	NA	NA	NA	NA
snp_11004	2840447	SPA2740	A/G	836	2	V/A	N
snp_30075	2895450	intergenic	T/C	NA	NA	NA	NA
snp_11034	2907408	SPA2807	T/C	225	3	L/L	S
snp_11037	2914086	SPA2813	C/G	943	1	G/R	N
snp_2980615	2980615	intergenic	A/G	NA	NA	NA	NA
snp_11073	3017733	SPA2908	C/G	624	3	T/T	S

SNP ID	Ref position	Gene	SNP	Pos in CDS	Pos in code	REF_AA/ Query_AA	SNP type
snp_11080	3033975	SPA2923	T/C	329	2	R/H	N
snp_3082867	3082867	SPA2977	C/T	1392	3	G/G	S
snp_3096125	3096125	SPA2995	C/T	88	1	E/K	N
snp_11111	3102029	SPA3001	A/G	661	1	F/L	N
snp_11160	3213511	SPA3107	A/G	261	3	G/G	S
snp_20152	3224028	intergenic	T/C	NA	NA	NA	NA
snp_11170	3237484	SPA3126	A/G	553	1	V/I	N
snp_11205	3306330	SPA3198	A/G	4346	2	R/K	N
snp_11214	3332785	SPA3225	T/C	291	3	I/M	N
snp_3340381	3340381	SPA3234	A/T	565	1	W/R	N
snp_11232	3370111	SPA3258	A/G	1317	3	Q/Q	S
snp_11240	3390915	SPA3282	T/C	409	1	V/I	N
snp_11245	3396706	SPA3294	T/G	381	3	N/K	N
snp_20167	3421953	intergenic	A/G	NA	NA	NA	NA
snp_3422038	3422038	SPA3332	G/A	4	1	V/M	N
snp_11276	3476592	SPA3377	T/C	477	3	G/G	S
snp_11278	3480749	SPA3379	T/C	1284	3	L/L	S
snp_11285	3498273	SPA3389	A/G	384	3	H/H	S
snp_20175	3556738	intergenic	T/C	NA	NA	NA	NA
snp_11317	3580287	SPA3467	A/G	486	3	T/T	S
snp_11325	3590982	SPA3473	T/C	967	1	A/T	N
snp_20179	3609824	intergenic	A/G	NA	NA	NA	NA
snp_3630574	3630574	SPA3507	C/T	138	3	L/L	S
snp_11353	3667705	SPA3541	T/C	51	3	D/D	S
snp_3873979	3873979	SPA3727	G/A	695	2	P/L	N
snp_11504	3910995	SPA3763	T/C	660	3	A/A	S
snp_11509	3924050	SPA3779	T/G	155	2	P/H	N
snp_3927385	3927385	SPA3781	T/C	151	1	Y/H	N
snp_11571	4079673	SPA3936	T/G	460	1	Q/K	N
snp_11599	4148576	SPA3994	A/G	93	3	N/N	S
snp_11631	4228720	SPA4071	T/C	2091	3	P/P	S
snp_11634	4233398	SPA4073	A/G	622	1	V/M	N
snp_4259353	4259353	SPA4085	G/T	1284	3	L/L	S
snp_11688	4376109	SPA4202	A/T	119	2	E/V	N
snp_11690	4377739	SPA4204	T/G	611	2	I/S	N
snp_20221	4395001	intergenic	A/G	NA	NA	NA	NA
snp_11725	4476471	SPA4306	T/C	2392	1	L/F	N

SNP ID	Ref position	Gene	SNP	Pos in CDS	Pos in code	REF_AA/ Query_AA	SNP type
snp_20227	4482274	intergenic	A/C	NA	NA	NA	NA
snp_4491551	4491551	SPA4315	C/T	415	1	G/S	N
snp_11775	4570173	SPA4395	T/C	350	2	P/L	N

Technical Appendix Table 3. The migration trend within the four provinces with the highest incidence of *S.* Paratyphi A infection from 1998 to 2012 (Data from National Bureau of Statistics of the People's Republic of China).

	2000 (The Fifth National Census)					2010 (The Sixth National Census)				
Place of Residence	Population size	Place of Birth				Population size	Place of Birth			
		Zhejiang	Guangxi	Guizhou	Yunnan		Zhejiang	Guangxi	Guizhou	Yunnan
Zhejiang	4,487,898	-	5332	28,882	6617	5,400,348	-	16,607	152,452	43,106
Guangxi	4,157,001	2834	-	6239	2524	4,362,551	3574	-	6011	2794
Guizhou	3,399,918	2767	4373	-	6907	3,332,265	7237	3960	-	6549
Yunnan	4,069,260	6271	3286	22,491	-	4,467,537	4763	3302	19,064	-

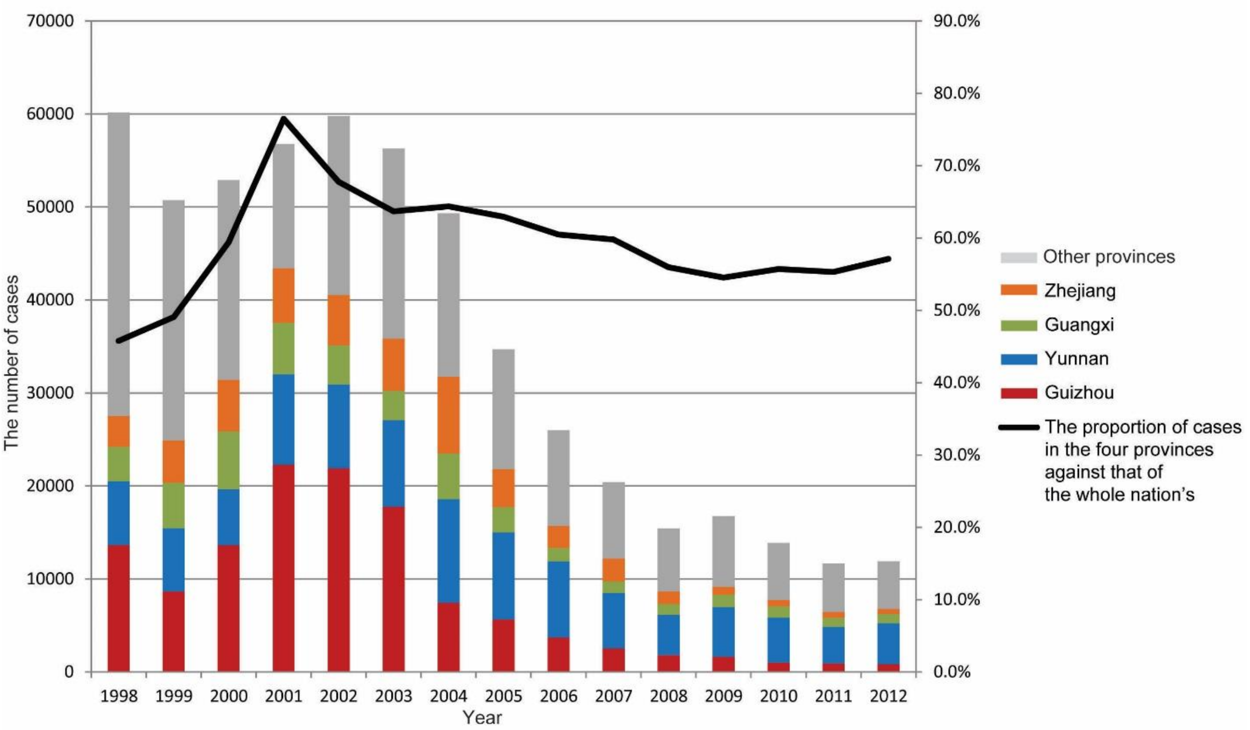

Technical Appendix Figure. Incidence of typhoid/paratyphoid fever in Guangxi, Guizhou, Yunnan, and Zhejiang and proportion of cases in these four provinces against that of the whole nation. The vertical bar plot indicates the numbers of cases of enteric fever in the four provinces with the highest incidence from

1998 to 2012. The bars are colored according to the inferred location, as shown in the legend on the right.

The curve shows the proportion of cases in all four provinces against that of the whole nation.
